# Variation in the oxytocin receptor gene is associated with behavioral and neural correlates of empathic accuracy

**DOI:** 10.3389/fnbeh.2014.00423

**Published:** 2014-12-05

**Authors:** Helle Ruff Laursen, Hartwig Roman Siebner, Tina Haren, Kristoffer Madsen, Rikke Grønlund, Oliver Hulme, Susanne Henningsson

**Affiliations:** ^1^Danish Research Centre for Magnetic Resonance, Centre for Functional and Diagnostic Imaging and Research, Copenhagen University Hospital HvidovreHvidovre, Denmark; ^2^Department of Neurology, Bispebjerg Hospital, University of CopenhagenCopenhagen, Denmark; ^3^Department of Neurorehabilitation TBI Unit, Copenhagen University Hospital GlostrupGlostrup, Denmark; ^4^Department of Clinical Biochemistry, Copenhagen University Hospital HvidovreHvidovre, Denmark; ^5^Center for Integrated Molecular Brain Imaging, Copenhagen University Hospital RigshospitaletCopenhagen, Denmark

**Keywords:** empathy, fMRI, 5-HTTLPR, oxytocin, serotonin

## Abstract

The neuromodulators oxytocin and serotonin have been implicated in regulating affective processes underlying empathy. Understanding this dependency, however, has been limited by a lack of objective metrics for measuring empathic performance. Here we employ a novel psychophysical method for measuring empathic performance that quantitatively measures the ability of subjects to decode the experience of another person's pain. In 50 female subjects, we acquired functional magnetic resonance imaging data as they were exposed to a target subject experiencing variable degrees of pain, whilst performing an irrelevant attention-demanding task. We investigated the effect of variation in the oxytocin receptor gene (*OXTR*) and the serotonin transporter gene (*SLC6A4*) on the psychophysical and neurometric variability associated with empathic performance. The *OXTR* rs2268498 and rs53576 polymorphisms, but not the *SLC6A4* 5-HTTLPR, were associated with significant differences in empathic accuracy, with CC- and AA-carriers, respectively, displaying higher empathic accuracy. For *OXTR* rs2268498 there was also a genotype difference in the correlation between empathic accuracy and activity in the superior temporal sulcus (STS). In *OXTR* rs2268498 CC-carriers, high empathic accuracy was associated with stronger responsiveness of the right STS to the observed pain. Together, the results show that genetic variation in the *OXTR* has significant influence on empathic accuracy and that this may be linked to variable responsivity of the STS.

## Introduction

Empathy refers to the capacity for feeling or understanding the experience of others, and is ubiquitous for adaptive social exchange. As a psychological trait, it varies significantly across the population (Baron-Cohen et al., 2001; McDonald et al., 2003) and is highly heritable (Davis et al., 1994), suggesting that genetic variation underpins at least some of the observed heterogeneity. From this perspective, the oxytocinergic and serotonergic systems are likely candidates for the expression of this heterogeneity; several studies have shown that manipulation of these systems have systematic effects on affective and social behavior (Harmer et al., 2003; Bhagwagar et al., 2004; Del-Ben et al., 2005; Anderson et al., 2007; Browning et al., 2007; Domes et al., 2007; Bartz et al., 2010; Guastella et al., 2010; Schulze et al., 2011). Regarding variation in the oxytocin receptor gene (*OXTR*) gene, two polymorphisms are of particular interest: rs53576 has been associated with variance in empathic performance in healthy populations, and is implicated in the etiology of autism (Wu et al., 2005; Lerer et al., 2008). This polymorphism, as well as rs2268498, has also been shown to influence limbic responses to socio-emotional stimuli (Tost et al., 2010; O'Connell, 2012). The rs2268498 has been associated with emotion recognition accuracy (Melchers et al., 2013), and, in combination with the 5-HTTLPR, an insertion/deletion polymorphism in the promoter region of the serotonin transporter gene (*SLC6A4*), it has shown a relationship to negative emotionality traits (Montag et al., 2011).

Regarding the neural underpinnings of empathic performance, brain regions implicated include those involved in emotion recognition, understanding of the beliefs and intentions of others (theory of mind; ToM) and social interaction, namely the amygdala, insula, anterior cingulate cortex (ACC), ventromedial and dorsolateral prefrontal cortices (PFC), temporal pole, temporo-parietal junction (TPJ), superior temporal sulcus (STS), precuneus, and posterior cingulate cortex (PCC) (Singer, 2006; Zaki et al., 2009; Decety, 2010; Xi et al., 2011; Zaki and Ochsner, 2012a). Amongst these regions, the amygdala, insula and ACC have also shown to be subject to serotonergic and oxytocinergic neuromodulation (Pezawas et al., 2005; Anderson et al., 2007; Arce et al., 2008; Petrovic et al., 2008; Tost et al., 2010; Riem et al., 2011).

There is a wide diversity of empathy metrics available, but most of these tests are limited by either (i) the fact that the target person's expressed experience was acted to produce the stimuli set or (ii) the target experience was real but the magnitude of the experience was not recorded (Baron-Cohen et al., 2001; McDonald et al., 2003). Thus in these cases there is no authentic experience of the target person to decode, and no benchmark from which to calculate empathic performance. One method (Zaki et al., 2009) has as stimulus a target person describing self-experienced life events and uses the self-rated experience as the objective truth, but the thoughts and feelings were reported by the target person retrospectively, thus making the reports liable to fallible memory and retrospective construal.

In the present study, we investigated behavioral and neural effects of genetic variation in the oxytocin receptor and serotonin transporter genes on empathic pain processing. The blood oxygen level dependent (BOLD) response to unattended facial expressions of acutely evoked pain was captured with fMRI. To avoid the above-mentioned limitations, the target person was video-recorded while experiencing and rating authentic pain. In order to prevent magnitude estimation and ratings of pain to influence empathic brain responses, fMRI data were acquired as the participants were attending to an empathy-irrelevant task superimposed on the pain expression video, whereas their ratings of the target person's pain were recorded separately. This provided two psychophysical measures of empathic performance: (i) empathic sensitivity, which measures the sensitivity of the subject to changes in pain in the target and (ii) empathic accuracy, which measures how close the subjects sensitivity is to the optimal decoding of the target person's pain. We expected both empathic sensitivity and accuracy to be influenced by emotion recognition and ToM abilities. Both oxytocin and serotonin have been shown to modulate brain activity also to unattended emotional expressions (Dannlowski et al., 2008; Kanat et al., 2014). Due to the association with emotional and empathic processing, we specifically tested for the effects of (i) the rs2268498 polymorphism in the promoter region of the *OXTR* gene, (ii) the rs53576 polymorphism in intron 3 of the *OXTR* gene, and (iii) the 5-HTTLPR polymorphism in the promoter region of the *SLC6A4* gene. We hypothesized that these polymorphisms would explain variability in empathic performance as well as in the underlying brain responses elicited by the unattended perception of pain expressions.

## Materials and methods

### Participants

We screened 94 healthy young women by advertisements posted on the internet. In order to avoid stratification due to possible gender differences in emotional responding, only females were included. Fifty of these met the genotypic inclusion criteria [see *Polymorphisms* below, mean age (*SD*) = 24.9 (4.9)]. These 50 participants were scanned between day 5 and 12 of the menstrual cycle, with the exception of four subjects who reported irregular periods. This was to avoid potential noise introduced by differences in emotional processing across the menstrual cycle (Derntl et al., 2008; Andreano and Cahill, 2010). Exclusion criteria included a history of antipsychotic or antidepressant treatment, and any diagnosis of affective disorder. The study was double-blinded, i.e., the participants as well as the person performing the scanning were blinded to the genotype of the participants. The study was approved by the local Ethics Committee of the Capital Region of Denmark (H-3-2012-121) and was conducted in accordance with the Declaration of Helsinki. Written informed consent was obtained from all participants.

### Polymorphisms

Participants provided saliva samples, and genotype status for *SLC6A4* 5-HTTLPR, *OXTR* rs2268498, and *OXTR* rs53576 was determined. The choice of polymorphisms was motivated by previous findings showing a relationship to emotional processing and empathy-related traits (Hariri et al., 2002; Harmer et al., 2003; Bhagwagar et al., 2004; Del-Ben et al., 2005; Pezawas et al., 2005; Wu et al., 2005; Anderson et al., 2007; Browning et al., 2007; Domes et al., 2007; Lerer et al., 2008; Rodrigues et al., 2009; Bartz et al., 2010; Guastella et al., 2010; Tost et al., 2010; Antypa et al., 2011; Schulze et al., 2011; Székely et al., 2011; Montag et al., 2011; O'Connell, 2012). The two *OXTR* polymorphisms are situated in the promoter region and intron 3, respectively, and are part of the same haplotype block (Campbell et al., 2011). The *OXTR* rs2268498 and *OXTR* rs53576 polymorphisms are in moderate linkage disequilibrium; several haplotypes show high frequencies and other research groups have found associations for one polymorphism in the haplotype block while another polymorphism did not show such an effect (Wu et al., 2005; Wermter et al., 2010; Walum et al., 2012), thus justifying the inclusion of both in the current study. The *SLC6A4* 5-HTTLPR genotype was classified as homozygous for the 5-HTTLPR short allele (SS) [also including the G variant of the rs25531 of the long allele (LL) which is apparently equivalent to the low-expressing S allele with regard to gene expression (Hu et al., 2006)] or homozygous for the LL. Individuals with heterozygous (SL) genotype (*n* = 44) were not included in the study due to unclear findings regarding serotonin transporter availability in individuals carrying this genotype (Little et al., 1998; Hu et al., 2006), resulting in a sample size of 50 subjects (see *Participants* above). For genotyping methods, see Supplementary Material 1.1.

### Experimental design

#### Stimulus material

**Figure 1:**
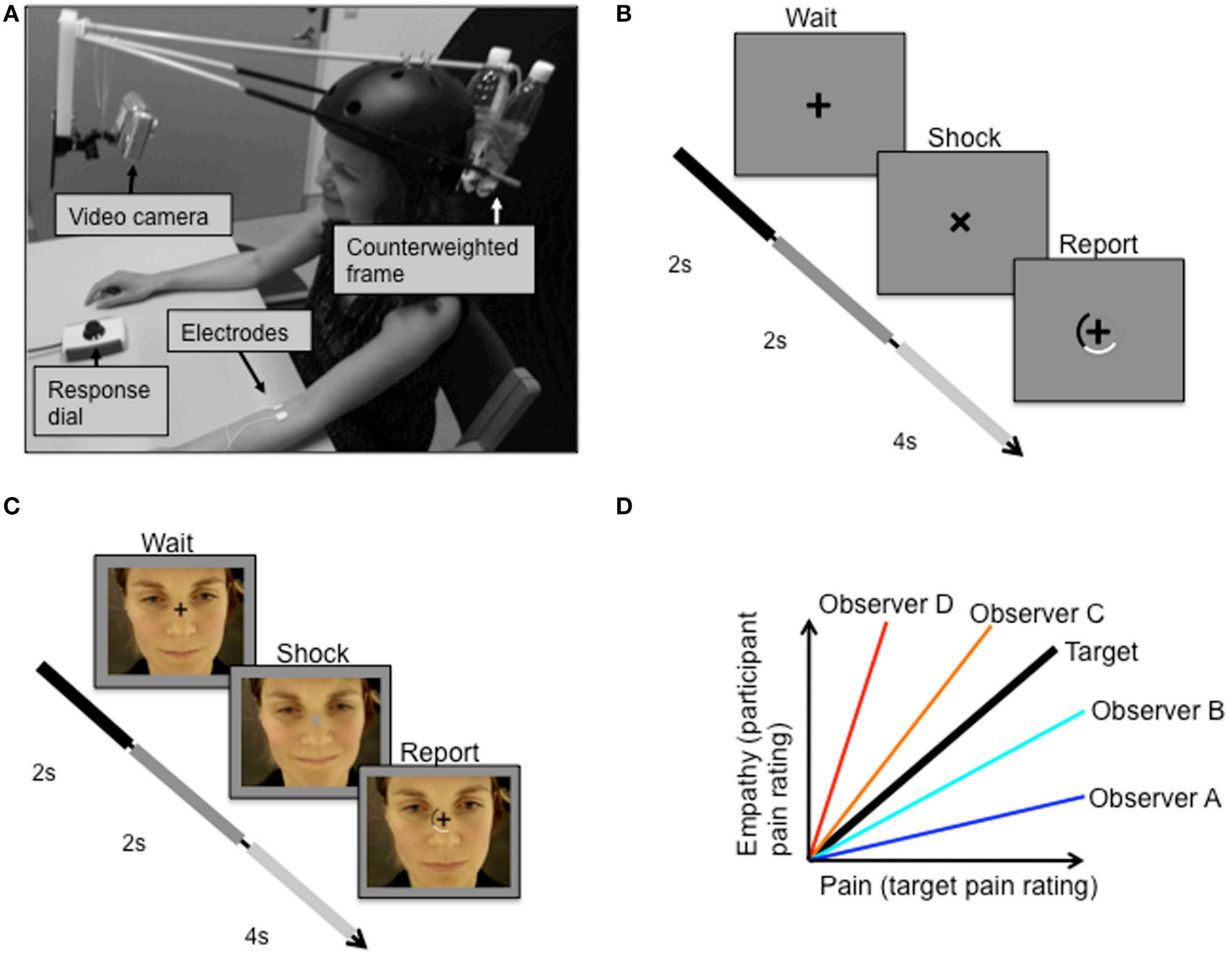
**Experimental protocol. (A)** Target received pulses to the left forearm while facial expressions were video recorded. **(B)** Target was instructed to fixate the cross on the screen in front of her, which rotated 45 degrees simultaneously with each electric shock. **(C)** Observer subjects were instructed to fixate a centrally presented fixation cross that was superimposed over the pain video. **(D)** The relationship between the target's pain ratings (Pain) and the observers' ratings of the target's pain expressions (Empathy). The bold black line represents perfect decoding of the target's pain ratings, i.e., veridical empathy. Two measures of empathy were of special interest: sensitivity and accuracy. Sensitivity is defined as the slope of the pain-empathy function, such that observers A and B are hyposensitive and observers C and D are hypersensitive. Empathic accuracy is defined as the distance between the veridical empathy slope and the observer's pain-empathy function slope; equal accuracy is illustrated for hypothetical observers such that observer A = observer D < observer B = observer C.

Two videos of a female experiencing electric stimulation to the arm of varying degrees of pain were shown to the participants (for video making methods, see Figures 1A,B, and Supplementary Material 1.2). These pain videos showed the facial responses of the female target person to electric stimulation (Figure 1C) that was applied at an inter-stimulus interval of 8.3 s (jitter: ±0.9 s). In each of the two videos, 180 electric pulses were applied to the target person. Repeated stimulation covered the whole range of intensities ranging from the target person's pain threshold (defined as the point at which pain begins to be felt) to pain tolerance threshold (defined as the maximum intensity that the target subject was willing to accept). Electric stimuli were given randomly and were not reported to the target subject. The two videos were identically recorded except for the order in which different magnitudes of electric stimulation were applied. Using a response dial, the target subject rated the magnitude of the experienced pain in each trial on a semi-circular visual analog scale. The response dial could be rotated in either direction to indicate the response magnitude on the screen, in relation to the two end points “no pain” and “worst pain imaginable.” The target subject was not informed of the purpose of the videos.

#### Procedure

Using standardized verbal and written instructions, participants were informed that they would watch a video depicting a female receiving electric stimulation to the forearm resulting in varying degrees of pain experiences. Participants viewed two different versions of these pain videos in the scanner (randomly ordered across subjects). Subjects were instructed to fixate a centrally presented fixation cross that was superimposed over the pain video. At times exactly simultaneous to the appliance of electric shocks to the target, the fixation cross would rotate by 45° for 2 s and change luminance for this duration. After 2 s the fixation cross rotated back, and a rating scale appeared for 4 s, during which participants were to report the lightness of the fixation cross (Figure 1C). After the presentation of the first video and just prior to the second video, participants were instructed to report the magnitude of pain experienced by the target subject during the second video, rather than the fixation cross luminance. Participants were informed that they would be rewarded money to the degree that they were accurate in judging the target person's pain. The monetary incentive, as well as the avoidance of mentioning the word empathy in the information to the participants, were employed to prevent an influence on the ratings from psychological mechanisms related to giving the impression of being an empathic, compassionate person. The scale and the slider box used by the participants were the same as those used by the target person when recording the pain videos. The participants' ratings of the pain expressions of the second video enabled determination of measures of empathic performance, including those described below.

In addition to the pain rating task in the scanner, participants also performed an emotion recognition test of static faces (see Supplementary Material 1.3.) outside of the scanner.

### Behavioral data analysis

#### Definitions of empathic performance measures

Veridical empathy, in which a subject could perfectly decode the experience of the target, would result in a pain-empathy function (i.e., pain as in the target's pain ratings, and empathy as in the participant's rating of the target's pain expression) of slope 1 with 0 variance (black line, Figure 1D). Based on the target's pain experience and the observer's empathic judgments of the target person's pain, we derived two objective measures of empathic performance, *empathic sensitivity*, and *empathic accuracy*, which are illustrated in Figure 1D. We define empathic sensitivity as the slope of the pain-empathy function, such that slopes steeper than veridical empathy (>1) represent empathic hypersensitivity, which would mean overly high estimates of pain inferred (observer C and D in Figure 1D), and the converse, hyposensitivity for slopes below (observer A and B in Figure 1D). Empathic accuracy is a symmetrical measure around the veridical empathy slope, defined as the slope when the slope is <1 (< veridical empathy slope) and as 1/slope when the slope is >1 (> veridical empathy slope). Accordingly, empathic accuracy is the same for a hypersensitive and a hyposensitive person with equal distance between their pain-empathy functions and the veridical empathy function, see observer B and observer C in Figure 1D. For slopes < 1, as sensitivity increases, so does accuracy, and, for slopes >1, increased sensitivity implies lower accuracy. Notably, a difference between groups in mean sensitivity implies a difference also in mean accuracy, while the opposite is not true.

#### Statistical analysis

Analyses of behavioral data were carried out in SPSS (version 20, Chicago, Illinois, USA). The statistical threshold for significance was set at *p* < 0.05.

### Functional magnetic resonance imaging

#### Image acquisition

Subjects were scanned using BOLD fMRI in a Siemens Magnetom Trio 3T MR scanner. Data were acquired with a gradient echo EPI sequence (repetition time (TR) = 2490 ms, echo time (*TE*) = 30 ms, matrix = 64 × 64, voxel size = 3 × 3 × 3 mm, volumes = 310, slices = 42, with a sequential slice acquisition order).

#### Imaging data analysis

The fMRI data were preprocessed and analyzed using the statistical parametric mapping software package (SPM8, http://www.fil.ion.ucl.ac.uk). Preprocessing included spatial realignment, slice timing correction, co-registration between functional and anatomical images, and normalization to the standard Montreal Neurological Institute (MNI) template and smoothing using a symmetric 8-mm Gaussian kernel.

The task was designed so that the pain response events could be estimated independent from the confounding effects of magnitude estimation and reporting. The following analyses focus on data from the first session where participants had the psychophysical task of reporting the lightness of the fixation cross. In the first session observers did not yet know that they would later be asked to rate the pain felt by the target person, thus the brain responses evoked by the pain expression videos are not influenced by task incentives. Pain responses were constructed using a stimulus onset regressor (time-locked to the onset of the target's shock) together with 1st order parametric regressors modeling (i) the target's pain ratings for that trial, (ii) the luminance of the fixation cross, and (iii) the observers lightness report. In addition, the subject-level models included 24 nuisance regressors to correct for movement artifacts, including 1st and 2nd order movement parameters and spin history effects. Low-frequency noise was removed by applying a high-pass filter (cut-off of 128s) to the fMRI time series at each voxel. Significant hemodynamic changes for each regressor and their parametric modulation were calculated based on the general linear model, and effects were convolved with the canonical hemodynamic response function (Friston et al., 1995).

Both the main effect of stimulus onset and the main parametric effect of target pain were then included in a random effects group-level analysis. Random effects analysis focused on the detection of brain regions where BOLD activity linearly increased with the target person's ratings of experienced pain (main effects of target's pain ratings in **Figure 3A**), i.e., the linear effect of target pain, and BOLD activity elicited by pain onset for the target irrespective of pain strength and rating (main effects of target pain onset in **Figure 3B**). Mean empathic accuracy for each participant was added as a covariate to the above-mentioned models. This enabled us to identify brain regions displaying a relationship between individual empathic accuracy and regional BOLD activity increasing with, either the target's pain ratings, or at target pain onset irrespective of pain strength. Flexible factorial models were used to identify differences in BOLD activity correlating with the target's pain expressions between 5-HTTLPR genotypes (SS and LL), rs2268498 genotypes (CC vs. TT, C vs. TT and CC vs. T), and rs53576 genotypes (AA vs. GG, A vs. GG and AA vs. G). Genotype differences in BOLD activity elicited by the onset of target pain (irrespective of strength) were investigated using the same models. Flexible factorial models were also used to analyse genotype differences in the relationship between empathic accuracy and BOLD activity elicited by increasing target pain ratings, and genotype differences in the relationship between empathic accuracy and BOLD activity elicited by target pain onset irrespective of pain strength. All activations are reported at cluster-level in standard MNI stereotactic space. We set the significance level for activated voxels at *p* < 0.05 corrected for multiple comparisons using the family-wise error correction at cluster level. The cluster extent threshold was set to *p* < 0.001 (uncorrected for multiple testing) and was used to illustrate the main effects in **Figures 3A,B**. Based on prior evidence showing that *OXTR* and *SLC6A4* variation affect amygdala activity during socio-emotional processing (Hariri et al., 2002; Tost et al., 2010) and linking insula and the ACC to empathy for pain (Hein and Singer, 2008; Lamm et al., 2011), we were particularly interested in the effect of the mentioned polymorphisms on activity in the amygdala, insula and ACC. We therefore restricted the correction for multiple comparisons to these *a priori* regions of interest (ROIs), as defined by PickAtlas (Maldjian et al., 2003). After ROI analyses, whole-brain analyses were performed, using whole-brain correction for all other brain regions.

## Results

### Genotypes

The genotype distributions for the *OXTR* genotypes did not differ significantly from Hardy Weinberg equilibrium (rs2268498: *p* = 0.38, rs53576: *p* = 0.97). They are displayed in Table 1, also stratified on 5-HTTLPR genotype. Consistent with previous reports of linkage disequilibrium between the two *OXTR* polymorphisms (Campbell et al., 2011), Table 1 shows that out of eight rs2268498 CC-carriers, as much as six (75%) were also rs53576 AA-carriers, and out of the 22 rs53576 GG-carriers, only one (5%) was a rs2268498 CC-carrier.

**Table 1:** **The genotype distributions for the *OXTR* and *SLC6A4* genotypes**.

			***OXTR* rs53576 AA**	***OXTR* rs53576 AG**	***OXTR* rs53576 GG**	***OXTR* rs53576 AA, AG, and GG**
5-HTTLPR SS	*OXTR* rs2268498	CC	5	1	1	7
		CT	1	5	5	11
		TT	0	2	6	8
		CC, CT, TT	6	8	12	26
5-HTTLPR LL	*OXTR* rs2268498	CC	1	4	0	5
		CT	1	7	4	12
		TT	0	1	6	7
		CC, CT, TT	2	12	10	24
5-HTTLPR SS and LL	*OXTR* rs2268498	CC	6	5	1	12
		CT	2	12	9	23
		TT	0	3	12	15
		CC, CT, TT	8	20	22	50

### Behavioral results

#### Empathic sensitivity

There were no genotype group differences in empathic sensitivity [*OXTR* rs2268498: *F*_(2, 47)_ = 0.873, *p* = 0.425; *OXTR* rs53576: *F*_(2, 47)_ = 1.422, *p* = 0.251; *SLC6A4* 5-HTTLPR: *t*_(48)_ = −0.763, *p* = 0.449] (see Supplementary Table 1 for mean empathic sensitivity for the different genotype groups).

#### Empathic accuracy

**Figure 2:**
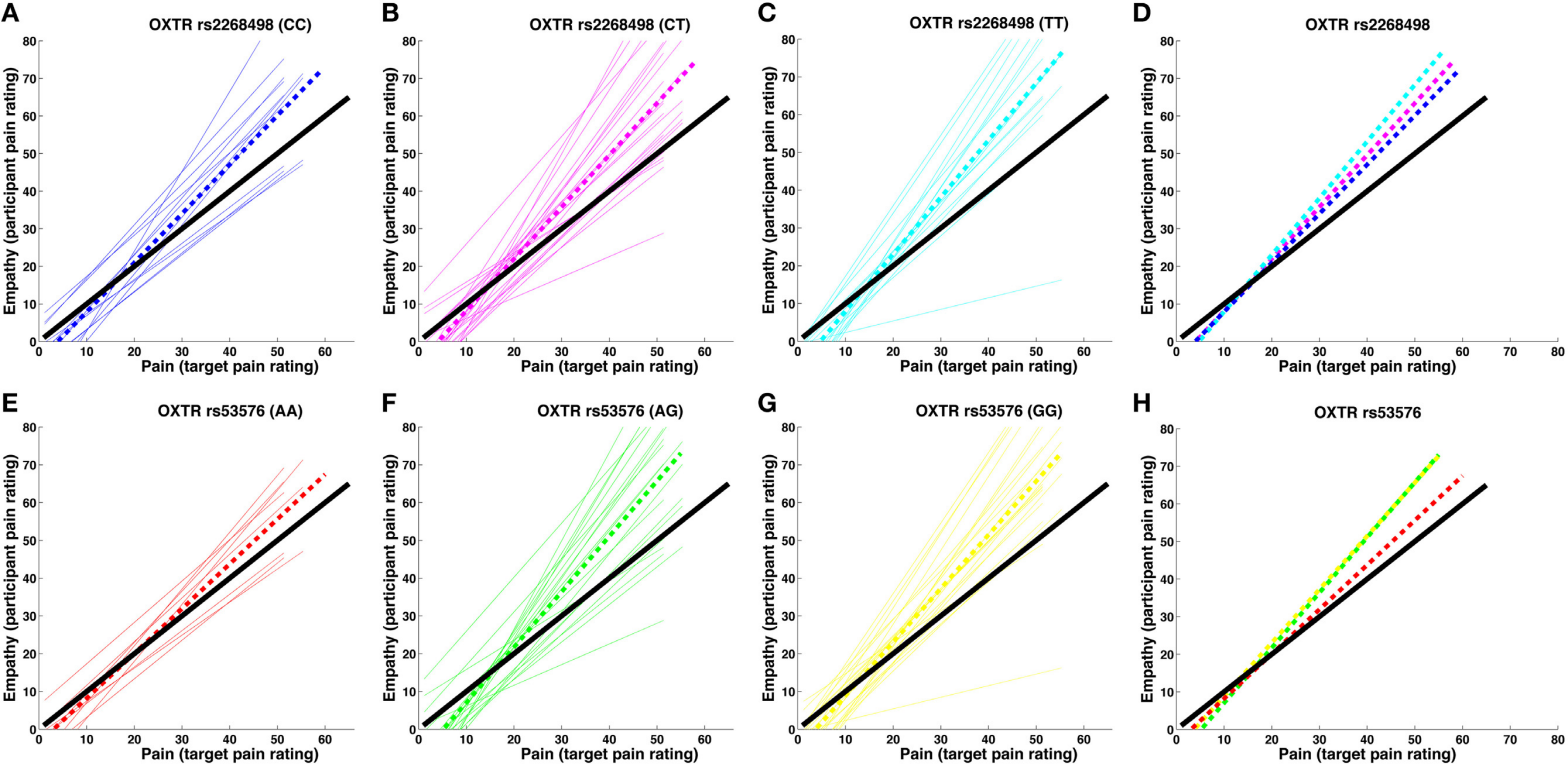
**Pain-empathy relationship for *OXTR* genotypes. (A–D)** show the pain-empathy relationship for rs2268498 genotypes: the dotted blue, magenta, and cyan lines represent the mean pain-empathy slope for carriers of the CC, CT, and TT genotypes, respectively. **(E–H)** show the pain-empathy relationship for rs53576 genotypes: the dotted red, green, and yellow lines represent the mean pain-empathy slope for carriers of the AA, AG, and GG genotypes, respectively.

*OXTR* rs2268498 and rs53576 genotypes showed significant differences in empathic accuracy [*OXTR* rs2268498: *F*_(2, 47)_ = 3.581, *p* = 0.036; *OXTR* rs53576: *F*_(2, 47)_ = 3.430, *p* = 0.041), with CC and AA genotypes displaying the highest accuracy measures (Figure 2). For *OXTR* rs2268498, *post-hoc* comparison of the homozygote genotype groups confirmed the presence of significantly higher accuracy scores in CC-carriers as opposed to TT-carriers [*t*_(25)_ = 2.581, *p* = 0.016]. The differences between CC- vs. CT- and CT- vs. TT-carriers were not significant [*t*_(33)_ = 1.354, *p* = 0.185, *t*_(36)_ = 1.685, *p* = 0.101]. For *OXTR* rs53576, *post-hoc* comparison confirmed the presence of significantly higher accuracy scores in AA-carriers relative to both GG- [*t*_(28)_ = 2.578, *p* = 0.015] and AG-carriers [*t*_(26)_ = 2.386, *p* = 0.025]. The effects were still significant after exclusion of the two participants (Figures 2B,C) displaying the lowest sensitivity [rs2268498 CC vs. TT: *t*_(24)_ = 2.350, *p* = 0.031; rs53576 AA vs. GG: *t*_(27)_ = 2.562, *p* = 0.016 and AA vs. AG: *t*_(25)_ = 2.251, *p* = 0.033]. There was no difference in empathic accuracy between rs53576 AG- and GG-carriers [*t*_(40)_ = 0.018, *p* = 0.986], nor between SS and LL carriers of the *SLC6A4* 5-HTTLPR [*t*_(48_ = 1.005, *p* = 0.320; see Supplementary Material Table 1 for mean empathic accuracy for the different genotype groups]. Given this effect of *OXTR* variation on empathic accuracy, we focused our imaging analyses on empathic accuracy and inter-individual variance in regional BOLD response that could account for the behavioral effect. There was no influence of genotype on recognition of static emotional faces in an emotional face recognition test (EFRT), and no correlation between empathic sensitivity or accuracy and recognition accuracy of static emotional faces (see Supplementary Material 2.1 for the EFRT).

### Functional magnetic resonance imaging results

#### Brain activity evoked by target pain

**Figure 3:**
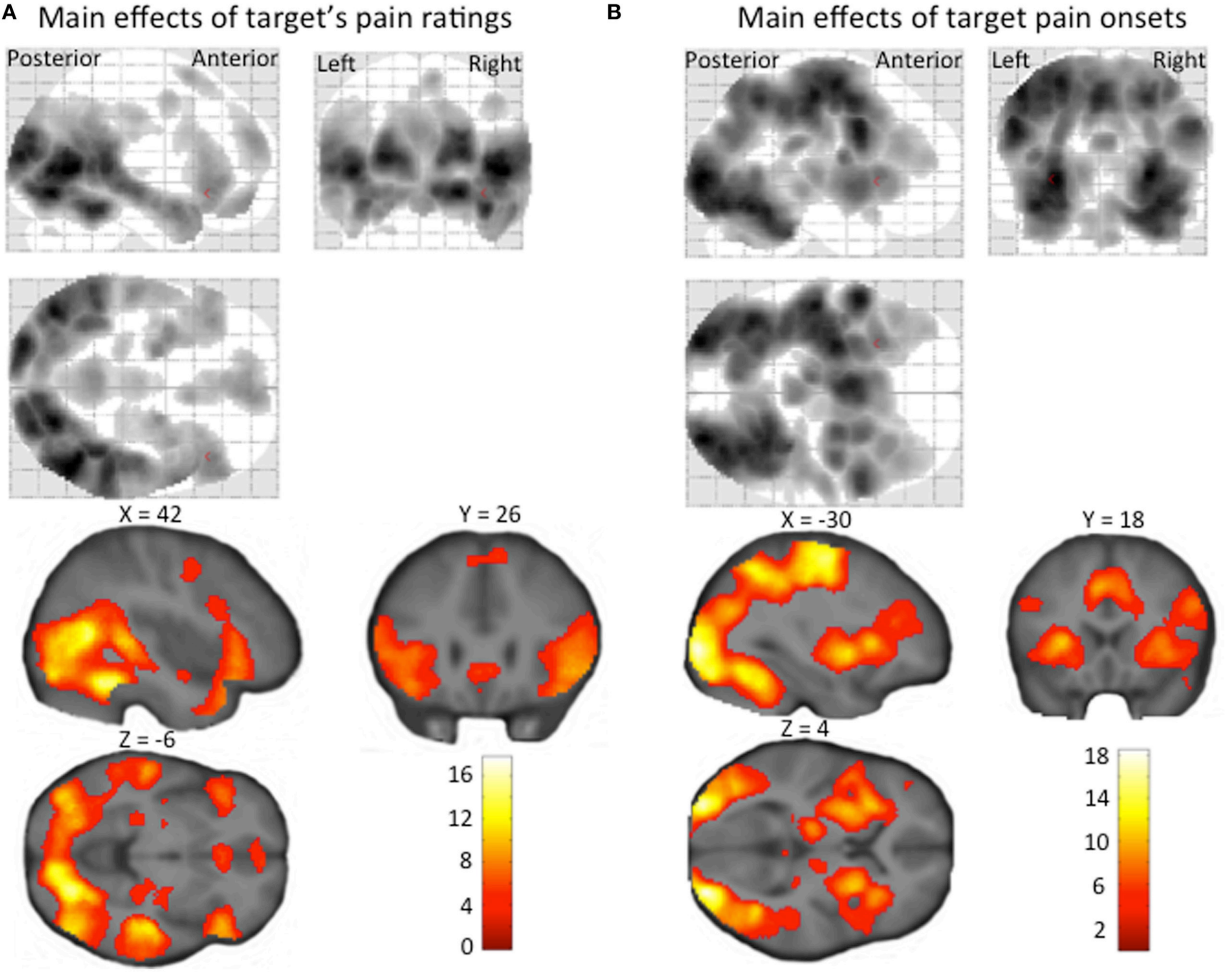
**Statistical parametric maps showing (A) a linear increase in BOLD signal with target's pain ratings, and (B) regional increases in BOLD signal at the time of target pain onset (irrespective of the pain intensity experienced by the target person)**. The maps are thresholded at an uncorrected *p*-value of *p* < 0.001.

Regions showing a positive linear effect of target pain included dorsolateral and ventromedial PFC, amygdala and ACC, as well as STS, TPJ, temporal pole, precuneus, and PCC (Figure 3A, Supplementary Material Table 2A). Regions correlating with the onset (orthogonalized on pain magnitude) of target pain comprised frontal, temporal, parietal and occipital activity, and activity in our *a priori* regions of interest: the anterior insula, amygdala, and ACC (Figure 3B, Supplementary Material Table 2B). In none of these regions, for neither the linear effect of target pain, nor the onset of pain, was there an effect of inter-individual differences in empathic accuracy.

#### Influence of genotype on the brain's response to target pain

For the *SLC6A4* 5-HTTLPR genotype, LL-carriers showed a stronger linear effect of target pain in the posterior cerebellum, compared to SS-carriers (peak at MNI coordinates: *x*, *y*, *z* = 6, −50, −8; *Z* = 4.45). There was no brain region where the *OXTR* rs2268498 or the *OXTR* rs53576 genotype significantly influenced the linear effect of target pain. Neither *SLC6A4* 5-HTTLPR genotype nor *OXTR* rs2268498 and *OXTR* rs53576 genotypes were associated with brain activity elicited by the onset of target pain.

#### Influence of genotype on the relation between empathic accuracy and the neural response to target pain

**Figure 4:**
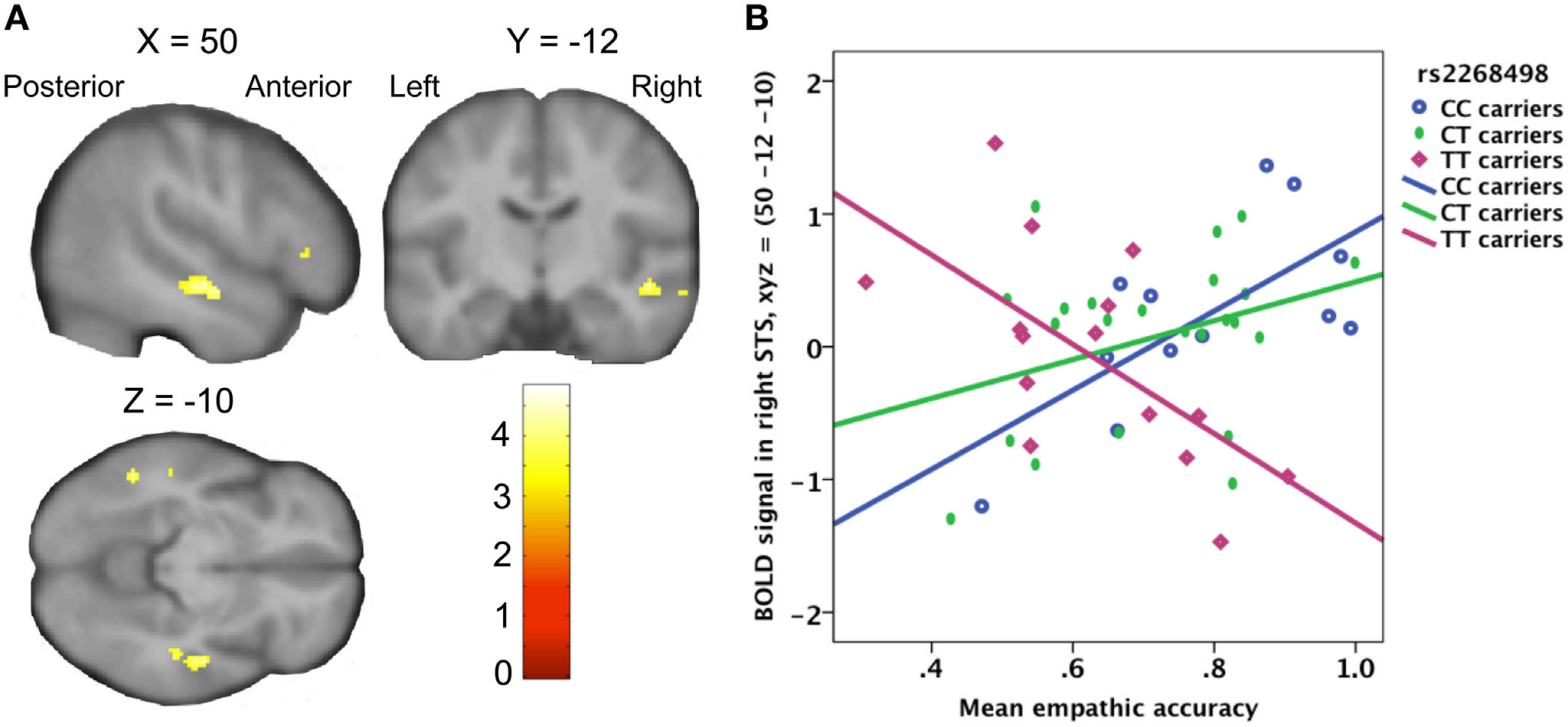
**(A)** Statistical parametric map showing a positive correlation between right STS activity and empathic accuracy for *OXTR* rs2268498 CC- and CT-carriers, compared to TT-carriers. The effect peaked at *x*, *y*, *z* = 50, −12, −10; *Z* = 3.95; *p* = 0.028). **(B)** Scatter plot of mean empathic accuracy and right STS. CC-carriers: *R*^2^ linear = 0.466; CT-carriers: *R*^2^ linear = 0.113; TT-carriers: *R*^2^ linear = 0.411.

There was no significant effect of genetic variation on the correlation between empathic accuracy and the brain response to the target's pain ratings. Variation in the *OXTR* gene did however have significant influence on the relation between the observer's empathic accuracy and the BOLD response to target pain onset in the right STS (peak at MNI coordinates: *x*, *y*, *z* = 50, −12, −10; *Z* = 3.95; Figure 4), irrespective of how strong the target person rated the pain. In CC- and CT-carriers, the BOLD response in right STS to painful face expressions positively correlated with empathic accuracy, whereas TT-carriers showed the opposite pattern (Figure 4B). The right STS expressed a similar effect for the *OXTR* rs53576 polymorphism, but this effect only reached trend significance (peak at MNI coordinates: *x*, *y*, *z* = −52, −30, −8; *Z* = 3.43). The AA- and AG-carriers showed a trend toward stronger STS BOLD response to painful face expressions with increasing empathic accuracy compared to GG-carriers (peak trend at MNI coordinates: *x*, *y*, *z* = 52, −16, −2; *Z* = 3.29). None of the *OXTR* polymorphisms showed a corresponding association with differences in the relationship between EFRT accuracy and BOLD response to painful facial expressions.

## Discussion

This study demonstrates a link between genotypic variation in the *OXTR* gene and individual differences in empathic accuracy while viewing painful facial expressions. Using a psychophysical measure of empathy that was based on the actual pain ratings by the target subject, we found that *OXTR* rs2268498 CC-carriers and *OXTR* rs53576 AA-carriers are more accurate when judging the pain experience of others. Using fMRI, we identified a potential mediator of these effects on accuracy, showing that the correlation between empathic accuracy and the neural response in STS to the onset of target pain depended on the *OXTR* genotype.

We also found that neural responses evoked by the unattended facial pain response of the target person scaled linearly to the pain magnitude experienced by the target person. A linear increase in regional activity with the magnitude of the target person's pain was observed in brain regions implicated in emotion recognition such as ventromedial PFC, amygdala and ACC (Phillips et al., 2003; Walter, 2012), as well as regions that have been related to ToM such as STS, TPJ, temporal pole, precuneus, dorsolateral PFC, and PCC (Xi et al., 2011; Walter, 2012; Zaki and Ochsner, 2012b). The activity profile in these regions discloses that both the emotion processing and ToM networks automatically and quantitatively encode the subjective distress of others. Notably, the correlation between activity and the target person's experience of pain was independent of magnitude estimation or reporting, since the target's pain expression was orthogonal to the distribution of physical and perceptual magnitudes invoked by the irrelevant perceptual task.

At the behavioral level, empathic accuracy was influenced by *OXTR* variation. The three *OXTR* rs2268498 genotypes showed significantly different empathic accuracy, with C-carriers being more accurate in their judgment of a target person's pain experiences. *OXTR* rs53576 genotypes also showed significant differences in empathic accuracy. The similar finding for the two polymorphisms is not surprising since they are in moderate linkage disequilibrium (Campbell et al., 2011). The association for the rs53576 should be compared to the study results of Rodrigues et al. (2009), showing that *OXTR* rs53576 influences dispositional and behavioral empathy as measured by a self-report empathy scale and the “Reading the Mind in the Eyes test” (Rodrigues et al., 2009). An association between the *OXTR* rs53576 and autism spectrum disorder has also been reported (Wu et al., 2005). However, while Rodrigues and colleagues showed A-allele-carriers to have lower behavioral and dispositional empathy, we found AA-carriers to be more accurate in their judgment of another individual's pain experiences, compared to AG- and GG-carriers. The psychophysical empathy test used here differs from the “Reading the Mind in the Eyes test” in two important aspects that may contribute to this discrepancy: Firstly, we measured the experience of the target person, and defined accuracy in relation to that rating. Secondly, it is specific to the dimension of pain only.

Since there was a general tendency for the participants to be hyper- rather than hyposensitive, the carriers of the genotypes associated with higher mean accuracy also had lower mean sensitivity (Figure 2). Notably, an increased hypersensitivity (slope larger than one), here related to a lower accuracy measure, may in other study designs be related to a better performance measure on emotion recognition and empathy. Meaningful comparisons are however, prevented since previous studies either did not use a design (Rodrigues et al., 2009; Melchers et al., 2013) or a measure (aan het Rot and Hogenelst, 2014) that could distinguish between sensitivity and accuracy. We acknowledge that different mechanisms may be involved in empathic accuracy and sensitivity and expect it more likely for empathic hypersensitivity to be influenced by a general negative bias, i.e., an exaggerated interpretation of negative emotions.

The tendency for hypersensitvity observed in all genotype groups reported here is interesting. However, one limitation of the present study is that by virtue of only testing subjects on one target person, we cannot infer whether hypersensitivity is a general feature of human empathic systems, or whether it is due to the idiosyncratic effect of one very expressive target subject. To infer on this we would need to repeat the test across numerous target subjects.

At the neural level, the central finding of this paper is the interaction between *OXTR* genotype and the expression of empathic accuracy in the STS. *OXTR* rs2268498 C-carriers, compared to TT-carriers, had a stronger positive correlation between the regional BOLD response in right STS and empathic accuracy, a relationship that was independent of the magnitude of the pain expression. This leads to the hypothesis that the enhanced empathic accuracy of *OXTR* rs2268498 C-carriers is mediated by its effects on the STS. It should be noted here, that the magnitude judgments underlying empathic accuracy assessment only occurred in the second session, and thus are from a dataset that is independent of the reported STS activity. Therefore, the influence of *OXTR* genotype on the relationship between empathic accuracy and the neural response of STS response to unattended pain expressions is not confounded by magnitude ratings of the target person's pain. The involvement of the STS in empathic processing is consistent with results reported by Zaki et al. (2009) showing the same region of the STS (MNI coordinates: 52 -10 -18) to be associated with a main effect of empathic accuracy, where accuracy is evaluated by comparison of participants' affective reports with the target's retrospective reports. More broadly, this effect is consistent with other studies showing posterior STS and oxytocin involvement in both ToM and emotion recognition (Singer, 2006; Domes et al., 2007; Heinrichs et al., 2009; Krämer et al., 2010; Schulze et al., 2011; Haas et al., 2013). *OXTR* variation did not influence the accuracy to recognize static faces expressing fear, anger, disgust or sadness (see Supplementary Material 2.1). It has been pointed out that biological motion is more effective in eliciting STS activity than are static pictures (Thompson and Parasuraman, 2012), possibly indicating that the use of video stimuli in the current study increased the engagement of this region.

It is worth noting that there are gender differences both in the STS engagement during empathic processing (Schulte-Rüther et al., 2008) and in the effect of oxytocin on emotional processing (Domes et al., 2007, 2010; Rilling et al., 2014). The results for the current sample of women should therefore not be generalized to men without further investigation. Methodological limitations including the low sample size warrant cautious interpretation of the current findings until replication attempts have been conducted, especially for negative findings such as those reported for sensitivity. Previous studies have found that the influence of oxytocin may differ between different subgroups, defined e.g., by low (Scheele et al., 2014) or high (Bartz et al., 2010) levels of autism traits. While the sample size of the current study did not allow for stratification on such measures, future studies should pursue this topic.

It should be noted that the functionality of the two *OXTR* polymorphisms tested here remains unknown. *OXTR* rs22468498 is located in the promoter region of the gene and the C allele is associated with higher mRNA expression, which is likely to have an effect on the number of oxytocin receptors (Montag et al., 2011). *OXTR* rs53576 is positioned in the third intron and has to date no known regulatory function. It therefore remains an open question whether another polymorphism in linkage disequilibrium with both rs2268498 and rs53576 is driving these effects. Future work should address how these two polymorphisms are related to oxytocin levels and oxytocinergic signaling in the brain, in order to better provide a biological context to the findings reported here.

There was no association between 5-HTTLPR and empathic performance, and also no effect of 5-HTTLPR on the relation between BOLD response and empathic sensitivity or accuracy. We did, however, find an association between the 5-HTTLPR and activity in the posterior cerebellum, with LL-carriers showing a stronger positive correlation between the target's pain ratings and BOLD response, compared to SS-carriers. Previous studies (Singer et al., 2004; Jackson et al., 2005) have found that the cerebellum is activated when perceiving and assessing painful situations, suggesting that this brain region is part of a “pain matrix.” However, this was not hypothesized a priori, and we leave it as an exploratory finding, worthy of future investigation.

## Conclusions

In conclusion, the subjects were highly accurate in decoding the subjective experience of pain from facial cues alone, and, even in the absence of attention to these cues, the subjective experience of others was quantitatively encoded in a diverse cortico-limbic network. There was significant inter-individual variation in empathic accuracy, and, we show that genetic variation in the *OXTR*, but not the *SLC6A4*, accounted for some of this inter-individual variability which was associated with differential evoked neural responses in the STS. The mechanisms mediating this putative oxytocinergic modulation of empathic accuracy, and its possible dysfunction in the empathic deficits of psychiatric disorder, thus remains an important and open challenge.

### Conflict of interest statement

The authors declare that the research was conducted in the absence of any commercial or financial relationships that could be construed as a potential conflict of interest.
