# Supplementary material for: Variation in the oxytocin receptor gene is associated with behavioral and neural correlates of empathic accuracy
Source: Front Behav Neurosci. 2014 Dec 5;8:423. doi: 10.3389/fnbeh.2014.00423 (PMC4257152; doi:10.3389/fnbeh.2014.00423)
Supplement: Supplementary file 1 [file Presentation1.PDF]

Supplementary Material

Variation in the oxytocin receptor gene is associated with behavioral and neural correlates of empathic accuracy

Helle Ruff Laursen[1], Hartwig Roman Siebner[1,2], Tina Haren[1,3], Kristoffer Madsen[1], Rikke Grønlund[4], Oliver Hulme[a], Susanne Henningsson[1,5] *

[1] Danish Research Centre for Magnetic Resonance, Centre for Functional and Diagnostic Imaging and Research, Copenhagen University Hospital Hvidovre, Denmark
[2] Department of Neurology, Bispebjerg Hospital, University of Copenhagen, Copenhagen, Denmark
[3] Department of Neurorehabilitation TBI Unit, Copenhagen University Hospital Glostrup, Denmark
[4] Department of Clinical Biochemistry, Copenhagen University Hospital Hvidovre, Denmark
[5] Center for Integrated Molecular Brain Imaging, Copenhagen University Hospital Rigshospitalet, Denmark

***Correspondence**
Susanne Henningsson, Danish Research Centre for Magnetic Resonance, Centre for Functional and Diagnostic Imaging and Research, Copenhagen University Hospital Hvidovre, Kettegård Allé 30, 2650 Hvidovre, Denmark.
Email: susanneh@drcmr.dk

1. Supplementary Methods

1.1. Genotyping methods

DNA was purified from saliva collected in Norgen saliva collection tubes (Norgen Biotek Corp., Toronto, Canada) using the Blood DNA purification kit on an automatic Maxwell 16 system (Promega). 5-HTTLPR genotyping was performed by restriction fragment length polymorphism (RFLP) method. The 5-HTTLPR promoter region was amplified by the primers reported in Wendland *et al.* 2006 resulting in a PCR product of 512 bp for the long allele and 469 bp for the short allele. PCR reactions were performed in a total volume of 25 µl with 50 ng DNA, 0.4 µM of each primer, 200 µM dNTPs, 1.0 Unit of Hot Start Taq polymerase (Ampliqon) and 1x ammonium buffer (Ampliqon). Amplification conditions were initially 15 min. at 95 °C followed by 35 cycles of 95 °C for 30s, 64 °C for 60s, 72 °C for 45s and finally 10 min. of terminal elongation at 72 °C. For identification of the rs25531 variant in the long allele (L_G), PCR products were digested with MSPI (Thermo Scientific) for 2 hours at 37 °C. This restriction endonuclease recognizes and cuts only when the G variant is present in the long allele, resulting in two fragments of 111 bp and 401 bp. PCR and RFLP products were analyzed with capillary gel electrophoresis (Fragment Analyzer™, Advanced Analytical). The two *OXTR* polymorphisms rs2268498 and rs53576 were genotyped using two commercially available Taqman SNP Genotyping Assays (LifeTechnologies/ABI). Briefly, 50 ng of DNA was mixed with 2x Taqman Universal PCR Master Mix and 40x SNP Genotyping Assay Mix in a total volume of 20 µl. The Allelic Discrimination Program on the ABI 7500 PCR system was used to determine the OXTR genotypes.

1.2. Video-making methods

In each of the two stimulation sessions, 180 electric pulses were applied to the target subject's left forearm at variable stimulus intensities using a constant current stimulator (Digitimer Ltd.,

Letchworth Garden City, UK). Stimuli were given every 8.3 s with a jitter of 0.9 s and split in six consecutive runs consisting of 30 stimuli. Electrical stimulation was applied through skin electrodes (Ambu Neuroline 700) were placed on the left forearm close to the elbow (Figure 0A). We used a bipolar stimulus arrangement with the two electrodes attached 2 cm apart. Electrical stimulation varied stochastically in current magnitude and ranged from individual pain threshold to the maximally tolerable stimulus intensity. The current magnitudes were randomly sampled from a flat probability distribution across this range, and were blinded from the subject. Pain threshold and tolerance limits were assessed before the experiment and were defined as the "minimum intensity at which the electrical stimulus was perceived as painful" and "the maximum intensity of a pain-producing stimulus that the target subject was willing to accept in a given situation", in accordance with a highly validated pain measurement method (Hjermstad et al. 2011). Target subjects were instructed to fixate the cross on the screen in front of them, which rotated 45 degrees simultaneously with each electric shock (Figure 0B). Two seconds after each electrical stimulation, as signalled by the rotation of the cross to its original orientation, the target subject was instructed to rate the magnitude of experienced pain on a visual analogue scale shown as a curved line on the screen using a response dial (PicoLog P216 Data Logger, Pico Technology), designed in order to minimise eye movements from fixation. The dial could be rotated in either direction to indicate the response magnitude on the screen, in relation to the two anchors "no pain" and "worst pain imaginable", in accordance with the pain metric (Hjermstad et al., 2011). To minimise sequential motoric and magnitude judgment effects, the gain, length, and starting orientation of the response magnitude graphic was randomised between trials. In between each run, the target subject rested for ~three minutes. Throughout the session, the target's facial expressions were recorded. Participants were seated and fitted with a head-mounted camera (Sony Cyber-shot 7.2 mega pixels) oriented frontally, with the field of view being centered on the bridge of the nose, and against the background of a photographic screen. This set up allowed for the collection of high-definition full facial video that precluded additional bodily information from the chin down, as well as confounding effects of stimulus induced head motion. The six videos that were recorded were shuffled in time and concatenated into two videos, one for each session. Eight trials were excluded resulting in two video clips per target subject. Each video clip contained 86 facial responses to electrical stimulation and lasted 12 min and 30 s.

1.3. Methods for Emotional face recognition test

Participants also performed an emotional face recognition test (EFRT). Pictures of eight nuances of different facial emotions, including fear, anger, disgust and sadness, were presented to the subjects, who indicated which of the emotions they thought the face was expressing by clicking with the mouse on the appropriate option. The pictures were taken from the FACES database (Ebner et al. 2010). To obtain different resolutions of the valence for the five emotional categories, each emotion was morphed with the neutral image of the same face identity, resulting in the following resolutions: 20, 30, 40, 50, 60, 70, 80 and 100% emotional valence with the lowest resolution only including 20% of the pixels from the emotional face image and 100% resolution being the emotional face image with no morphing.

2. Supplementary Results

2.1 Emotional face recognition test

EFRT-accuracy, *i.e.* the recognition of static aversive emotional faces, did not correlate with empathic sensitivity or empathic accuracy (rs2268498: correlation coefficient = -0.033, rs53576:

correlation coefficient = -0.085, *p*-values > 0.5). There was no significant difference between either *OXTR* or 5-HTTLPR genotypes in EFRT-accuracy (*OXTR* rs2268498: $F_{(2)} = 2.512$, $p = 0.092$; *OXTR* rs53576: $F_{(2)} = 2.763$, $p = 0.073$; 5-HTTLPR: $t = -0.602$, $p = 0.550$).

3. Supplementary Tables

Supplementary Table 1. Mean empathic accuracy and mean empathic sensitivity for the different genotype groups

		Empathic accuracy, mean (SD)	Empathic sensitivity, mean (SD)
OXTR rs2268498	CC	0.783 (0.163)	1.305 (0.350)
	CT	0.71 (0.147)	1.381 (0.419)
	TT	0.626 (0.153)	1.509 (0.442)
OXTR rs53576	AA	0.832 (0.127)	1.185 (0.244)
	AG	0.678 (0.163)	1.467 (0.456)
	GG	0.677 (0.151)	1.42 (0.402)
5-HTTLPR	SS	0.724 (0.138)	1.359 (0.353)
	LL	0.679 (0.181)	1.448 (0.468)

Supplementary Tables 2. fMRI main effects

Supplementary Table 2A. Main effects of parametric modulation with target's pain ratings

Area of activation	Side	*z*-value	*x*	*y*	*z*
Insula	R	6,34	42	26	-6
Insula	L	5,94	-36	26	4
Amygdala	R	6,93	-20	-8	-16
Amygdala	L	6,51	22	-6	-14
Anterior cingulate	R	5,06	2	30	-4
Anterior cingulate	L	4,99	-2	26	-6
Hippocampus	L	4,8	-26	-28	-8
Pulvinar	L	4,83	-22	-30	2
Medial orbitofrontal	R	6,78	4	52	-14
Medial orbitofrontal	L	6,19	-6	52	-12
Medial frontal gyrus	R	6,53	8	56	24
Middle temporal gyrus	R	$z > 7$	54	-64	*6*
Fusiform	R	$z > 7$	24	-74	-8
Supplementary motor area	R	5,91	6	14	62
Supplementary motor area	L	4,12	-6	22	54
Precentral	R	5,45	48	2	50
Middle occipital gyrus	L	$z > 7$	-46	-74	6

$p<0.05$ corrected for multiple comparisons using the family-wise error correction

Supplementary Table 2B. Main effects of target pain onset

Area of activation	Side	*z*-value	*x*	*y*	*z*
Insula	R	$z > 7$	-30	18	4
Insula	L	6,75	32	24	4
Amygdala	R	6,64	24	2	-12
Amygdala	L	6,21	-24	0	-12
Anterior cingulate	R	5,13	12	22	28
Posterior cingulate	R	$z > 7$	4	-34	24
Precentral	L	$z > 7$	-54	6	32
Middle occipital gyrus	R	$z > 7$	30	-92	4
Middle occipital gyrus	L	$z > 7$	-28	-94	2

$p<0.05$ corrected for multiple comparisons using the family-wise error correction

4. References

Wendland, JR., Martin, BJ., Kruse, MR., Lesch, KP. and Murphy, DL. (2006). Simultaneous genotyping of four functional loci of human SLC6A4, with a reappraisal of 5-HTTLPR and rs25531. *Mol Psychiatry*. 11(3):224–6.

Hjermstad, MJ., Fayers, PM., Haugen, DF., Caraceni, A., Hanks, GW., Loge, JH., et al. (2011). Studies comparing Numerical Rating Scales, Verbal Rating Scales, and Visual Analogue Scales for assessment of pain intensity in adults: a systematic literature review. *J Pain Symptom Manage*. 41(6):1073–93.

Ebner, NC., Riediger, M., Lindenberger, U. (2010). FACES - a database of facial expressions in young, middle-aged, and older women and men: Development and validation. *Behav Res Methods*. 42(1):351–62.
